# Risk factors associated with urinary metal concentrations in middle-aged and older Caribbean adults: the Tobago Health Study

**DOI:** 10.1038/s41370-026-00860-z

**Published:** 2026-03-30

**Authors:** Natalie F. Price, Ryan Cvejkus, Victor Wheeler, Patrick J. Parsons, Elizabeth J. Mullin, Chris Gennings, Aaron Barchowsky, Joseph M. Zmuda, Alison P. Sanders, Iva Miljkovic

**Affiliations:** 1 Department of Environmental and Occupational Health, University of Pittsburgh School of Public Health, Pittsburgh, PA USA; 2 Department of Epidemiology, University of Pittsburgh School of Public Health, Pittsburgh, PA USA; 3 Scarborough General Hospital, Scarborough, Trinidad and Tobago; 4 Division of Environmental Health Sciences, Wadsworth Center, New York State Department of Health, Empire State Plaza, Albany, NY USA; 5 Department of Environmental Health Sciences, College of Integrated Health Sciences, University at Albany, Albany, NY USA; 6 Department of Environmental Medicine, Icahn School of Medicine at Mount Sinai, New York, NY USA

**Keywords:** Metals, Biomonitoring, Dietary Exposure

## Abstract

**Background:**

Metal exposures are increasingly recognized as a major risk factor for chronic diseases, yet very few prior studies have comprehensively characterized metal concentrations in the Caribbean.

**Objective:**

This study characterized urinary metal concentrations and their associations with demographic characteristics and other lifestyle factors among middle-aged and older Tobagonian adults.

**Methods:**

We quantified urinary concentrations of 18 metals and metalloids (referred to here as “metals”) in 896 adults from a subsample of the Tobago Health Study, including 466 men and 430 women. Morning spot urine samples were collected from men in 2014–2016, and from women in 2019–2020. Metal concentrations were compared to studies of adults from other populations. Metal exposure risk factors included demographics, body composition, and lifestyle, including diet. We used sex-stratified partial least squares (PLS) regression to identify risk factors associated with metal concentrations.

**Results:**

Twelve metals including arsenic (As), barium (Ba), cadmium (Cd), cobalt (Co), cesium (Cs), copper (Cu), molybdenum (Mo), lead (Pb), tin (Sn), thallium (Tl), uranium (U) and zinc (Zn) were detected in >87% of participants in both men and women. Compared to other populations, Zn and Tl concentrations were higher among Tobagonian men and women. We observed distinct urinary metal concentration patterns among men and women that varied by age, education, body composition and lifestyle factors.

**SIGNIFICANCE:**

A mix of non-modifiable and modifiable risk factors associated with urinary metal concentrations were identified. These findings can inform future studies to identify vulnerable populations and tailor public health messaging to reduce exposure risk. Future research is needed to understand sex differences and how these exposures may contribute to adverse health outcomes.

**Impact:**

This study provides the first comprehensive characterization of urinary metal concentrations among middle-aged and older adults in Tobago, identifying both modifiable and non-modifiable factors associated with exposure. Twelve metals, including arsenic, cadmium, lead, and thallium, were detected in most participants, with some concentrations exceeding those reported in other populations. Distinct urinary metal concentration patterns that varied by demographics and lifestyle factors were observed among men and women. The exposure profiles of adults from Tobago can inform targeted public health strategies and lay the groundwork for future research on the health impacts of metal exposures in this understudied population.

## Introduction

Metals are naturally occurring and persistent in the environment, and low-level exposures among populations are unavoidable. Natural processes such as rock weathering, shifting ocean currents and forest fires lead to changing metal levels in the environment, and anthropogenic activities may exacerbate the release and transport of metals, bringing them into closer contact with human populations [1, 2]. In particular, resource extraction, manufacturing, untreated waste discharge, fossil fuel burning, and agricultural activities lead to the contamination of soil, water bodies, and air, which enables multiple pathways for population-based exposure through the ingestion of contaminated foods and water, inhalation of airborne particles, and direct contact in occupational settings [1]. The ubiquitous presence of environmental metals raises significant concerns about the potential human health risks associated with metal exposures, which include chronic conditions such as cardiovascular disease, metabolic syndrome, chronic kidney disease, neurological disorders, and cancer [3, 4].

Within a population, human biomonitoring demonstrates how changing environmental contamination is reflected in the metal concentrations in biological samples (e.g., blood, urine, hair) and provides a basis for studying the relationship between metal exposure and chronic disease trends [5]. Despite the importance of characterizing metal concentrations within a population, there is a significant lack of such data in Caribbean populations, limiting our understanding of how environmental contamination may affect these communities [6]. Prior studies in the Caribbean have assessed metal levels among vulnerable groups, including pregnant women and children [7]. However, there is a critical gap in understanding how metal exposures affect Caribbean populations selected regardless of health status, particularly middle-aged and older adults, who may have greater cumulative lifetime exposures, age-related physiological changes that influence metal toxicokinetics, and increased susceptibility to chronic diseases [8]. Because existing studies in the region have been cross-sectional and limited to certain populations, it remains unclear how metal exposure patterns may vary over time and contribute to the changing burden of chronic diseases. Establishing baseline biomonitoring data is therefore essential for detecting future changes in exposures and evaluating their potential health impacts in this population.

In addition to understanding how metal exposures vary across populations from different geographies, examining the potential risk factors that are associated with metal concentrations is vital. These factors, such as personal characteristics, lifestyle behaviors, and specific dietary patterns, can affect metal exposures and toxicokinetics. Identifying risk factors is an essential first step in identifying possible sources and pathways of exposure, assessing risk more accurately in subpopulations, and developing targeted strategies to reduce the health risks associated with metal exposures.

In this cross-sectional analysis of the Tobago Health Study (THS), we characterized urinary metal concentrations in a sample of 896 middle-aged and older African Caribbean adults. We compared metal concentrations to a nationally representative sample of US adults and other global populations and identified risk factors for metal exposure, including personal characteristics, lifestyle factors, and dietary patterns.

## Methods

### Study population

The Tobago Health Study (THS) has been described previously [9, 10]. Briefly, it is a population-based, prospective cohort study of middle-aged and older African Caribbean men and women. From 1999 to 2003, 3170 predominantly African Caribbean men aged 40 and older were recruited for a population-based prostate cancer screening for the first time on the island of Tobago, part of the dual-island country of Trinidad and Tobago. To be eligible, men had to be ambulatory, non-institutionalized, and not terminally ill. Approximately 60% of all age-eligible men on the island participated, and participation was representative of the seven island parishes (administrative boundary) on the island. Public service announcements and word of mouth were the main methods of recruitment. To establish a comparable cohort of Tobagonian women, recruitment began in 2019 using the same recruitment strategies, inclusion criteria, and protocols as in the men’s study. While quantitative metrics of representativeness are not available, the recruitment design and high rates of participation in our sample reasonably reflect the broader Tobagonian middle-aged and older African Caribbean adult population.

A random sample of 1000 individuals (500 men and 500 women) aged 40 years or older with available urine samples and pQCT measures of muscle composition was selected from the cohort to quantify urinary metal concentrations. Of the 1000 eligible participants, 896 (466 men and 430 women) had complete metal concentration and risk factor data (Fig. S1). Due to the differences in years of sample collection, urinary metals were analyzed separately for men and women. The study was approved by the University of Pittsburgh and the Tobago Ministry of Health and Social Services Institutional Review Boards. Informed consent was obtained at study enrollment.

### Urinary metal concentrations

Urine was selected as the biospecimen matrix because it is a non-invasive, cost-efficient collection method suitable for a large number of participants. Urine concentrations are measured as part of the biomonitoring protocols of many national surveys, such as the US NHANES, which enables comparison with other population-level exposure data. Urine concentrations are valid indicators of internal dose for several metals that are primarily eliminated via urine (e.g., As) and can be interpreted as markers of recent or ongoing exposures for metals not efficiently eliminated in the urine (e.g., Cd). Urine concentrations of essential metals, which play a role in human physiological functions in some forms, may reflect normal excretion, excess exposure, or increased urinary loss due to dysregulated homeostasis. Accordingly, urinary concentrations of essential metals are not sufficient to infer nutritional adequacy or deficiency, nor to suggest any related health benefits or harms. For this reason, we report essential and nonessential metals together.

Morning spot urine samples were collected at the men’s sixth follow-up clinic visit from 2014–2016 (V6_men_) [11], and the women’s baseline visit from 2019–2020 (V1_women_) [10]. Metal and metalloid concentrations were measured in two 2-milliliter (mL) aliquots of urine. Urine samples were frozen at –80 °C and batch-shipped for permanent storage at the University of Pittsburgh. This approach ensured that the samples were preserved for subsequent analysis while maintaining their integrity without undergoing freeze-thaw cycles. The urinary metalloids (referred to here as “metals”) measured included arsenic (As) and antimony (Sb), and metals included barium (Ba), beryllium (Be), cadmium (Cd), cobalt (Co), chromium (Cr), cesium (Cs), copper (Cu), manganese (Mn), molybdenum (Mo), lead (Pb), platinum (Pt), tin (Sn), thallium (Tl), uranium (U), tungsten (W), and zinc (Zn). Of these, Co, Cr, Cu, Mn, Mo, and Zn are considered essential elements in certain forms; however, metal speciation was not conducted, and we were not able to distinguish between chemical forms that may differ in toxicity, bioavailability, or biological function.

The New York State Department of Health’s Wadsworth Center’s Human Health Exposure Analysis Resource (HHEAR), which provides laboratory expertise and targeted analyses to NIH-funded investigators, measured urinary trace metal concentrations for this study. The analytical methods were developed and validated specifically for use in the NIH CHEAR/HHEAR programs, and quality assurance protocols for HHEAR were followed for this study [12]. An Inductively Coupled Plasma Tandem Mass Spectrometer (Agilent Model 8900 ICP-MS/MS) was used to measure metal concentrations in urine. This state-of-the-art ICP-MS/MS system is equipped with an Octopole Reaction System (ORS) and axial acceleration technology, which was optimized for handling the multiple interferences arising from urine matrices. The system was configured with an SPS 4 autosampler (Agilent Technologies, Santa Clara, CA, USA) and an ultra-low particulate arrester air filter (Elemental Scientific, Omaha, NE, USA), which minimizes airborne contamination during each analytical run.

For analysis, thawed urine samples were mixed on a laboratory rocker, and a 0.3-mL aliquot was sampled for analysis by ICP-MS/MS. Urine samples were diluted to a 5-mL final volume using a reagent solution containing 2% (v/v) double-distilled nitric acid (HNO₃), 0.5% (v/v) double-distilled hydrochloric acid (HCl), 1000 µg L⁻¹ gold (Au) (Inorganic Ventures, Christiansburg, VA, USA), and 0.005% (v/v) Triton X-100™ (Sigma-Aldrich Co., St. Louis, MO, USA) in deionized (DI) water. To monitor method performance, each batch of urine samples was analyzed along with tri-level internal quality control (IQC) materials and HHEAR urine reference materials. The urine-based IQC materials comprised three different concentration levels (low, medium and high) covering the expected clinical range for the 18 trace elements measured. All three IQC levels were included at the start, middle and end of each analytical to ensure the method was operating under conditions of repeatability. This IQC procedure ensures that the analytical data are free of any egregious batch effects that could inflate measurement uncertainty. Method accuracy was periodically verified against Standard Reference Material (SRM) 2668 Toxic Elements in Frozen Human Urine (National Institute of Standards and Technology (NIST), Gaithersburg, MD) and found values remained within ± 20% of the assigned values, where established. All elevated results for each element were repeated and confirmed, and 2% of urine samples were randomly selected for duplicate analysis. External QA/QC included successful participation in six different External Quality Assessment Schemes (EQAS) and Proficiency Testing (PT) programs for trace elements in urine, including those operated by Le Centre de Toxicologie du Quebéc (CTQ), the UK Trace Elements Quality Assessment Scheme (TEQAS), The German External Quality Assessment Scheme (GEQUAS) for Trace Elements, The College of American Pathologists PT Program, The Centers for Disease Control and Prevention’s (CDC) PT program for the Laboratory Response Network, and the New York State Department of Health’s Biomonitoring PT Program for Trace Elements, which is operated by the Wadsworth Center’s HHEAR Trace Elements Laboratory. The NIH-supported HHEAR network has established a rigorous QA/QC program as described elsewhere [12]. The Wadsworth HHEAR laboratory’s internal and external QA/QC protocols are designed to ensure the high quality of the biomonitoring data for these studies.

Some metal measures were below the method limit of detection (LOD), and the ICP-MS/MS software-generated data were imputed as LOD/√2, as is common practice for dealing with left-censored data in environmental health studies [13]. Only those metals that were above the LOD in >70% of participants were included in the statistical analyses to ensure the robustness of statistical tests [14].

### Urinary creatinine measurement

Urinary creatinine was also measured to establish the participant’s hydration status. Urinary creatinine was measured by liquid chromatography tandem mass spectrometry (LC/MS/MS) using a well-validated method (Waters Acquity LC coupled to a Sciex API4000 MS/MS system). Samples were prepared for analysis by diluting urine samples 1000-fold with DI water and an isotopically labeled internal standard. Creatinine was separated from other matrix components on an Ascentis Express F5 column (100 × 2.mm, 2 µm) using an isocratic elution with 26:74 (5% methanol in water: acetonitrile—both supplemented with 0.1% formic acid). Quantitation was achieved using the stable-isotope dilution technique using creatinine standards in DI water. The geometric mean concentration of urine creatinine was 171 mg/dL. All but one value was above the method LOD (20 mg/dL), and its value was imputed as LOD/√2. These laboratory results were reviewed and approved according to the Wadsworth Center’s QA/QC policies to ensure that they conformed to acceptable quality standards [15]. Following guidelines from Barr et al [16], six samples (<1%) were identified as dilute (<30 mg/dL) and 110 (11%) as concentrated (>300 mg/dL). Across 18 assay batches, three internal QC pools (high, medium, and low) were analyzed with 100% validity and low coefficients of variation, i.e., repeatability (3–7%). All 64 blinded duplicate samples for creatinine were valid, with a median RPD of 2% and an ICC of 0.997. Additionally, the Wadsworth laboratory participates successfully in the Wisconsin and G-EQUAS PT programs for creatinine.

### Risk factor assessment: demographic, health behaviors, and lifestyle factors

Detailed information about potential risk factors was collected by questionnaire and/or physical examination administered by trained staff. Demographic characteristics included age, sex, and education. Education, which was collected as the highest grade level completed, comprised the completion of (1) primary school, (2) secondary school or technical vocational training, and (3) some university or higher. Health behaviors and lifestyle factors included smoking status and alcohol use. Smoking status was defined as ever smoked (current and former smokers) and never smoked. Alcohol use was defined as consuming more than four units of any type of alcohol in one week. Physical activity indicators included hours spent walking per week, watching television for more than 14 h per week, and sleep duration in average hours per night. A food frequency questionnaire captured the daily consumption of different foods in grams. The foods included in this analysis were fish, processed fish (including tinned and preserved fish), chicken, rice, non-starchy root vegetables, starchy root vegetables, and legumes. These foods were selected because they are commonly associated with metal exposure, either due to bioaccumulation of metals in meat from the animals’ consumption of contaminated food or through direct uptake from metal-contaminated soil in the case of plant-based foods [17]. Weight and height were collected and used to calculate BMI (kg/m^2^), which was included as a continuous variable. Muscle attenuation, which measures muscle quality by assessing fat infiltration, was calculated using peripheral quantitative computed tomography (pQCT) imaging. We performed pQCT scans of the lower leg using a Stratec XCT-2000 scanner. A trained technician conducted the scan following standardized procedures, which involved a cross-sectional image at 66% of the calf length of the left leg, as measured from the terminal end of the tibia. A trained investigator, unaware of the participants’ disease status, analyzed the images using STRATEC software version 5.5D (Orthometrix, Inc., White Plains, NY). Muscle attenuation is calculated by dividing muscle density (mg/cm) by muscle area (cm^2^). Lower attenuation values indicate a greater intramuscular adiposity content or increased fat infiltration within the muscle tissue.

### Statistical analysis

The distribution of urinary concentrations for highly detected metals (>70% of samples above LOD) was summarized by sex with the geometric mean and geometric standard deviation, as well as percentiles (minimum, 10th, 25th, 50th, 75th, 90th, maximum). Spearman’s correlation coefficients were calculated between each pair of highly detected urinary metal concentrations among men and women separately.

The geometric means for each measured metal were compared with study populations from 13 countries in the Americas, Europe, Africa and Asia [7, 18–25], as well as with a nationally-representative population of US adults from the National Health and Nutrition Examination Survey (NHANES) as this was the most comprehensive publicly available individual level data [26, 27]. Other studies that included middle-aged or older adult populations were included for comparison if they measured more than one metal, presented the population geometric means of unadjusted metal concentrations, and collected samples for metal measurement within five years of THS sample collection for both men and women. The NHANES population was stratified by men and women within the same age range as the THS population (40–87 years). Data from NHANES 2017–2018 were used for the comparison of 9 urinary metal concentrations, while U was collected during the 2015–2016 survey. Sampling weights were applied to make results nationally representative of the total US population, which equated to the following population sizes: NHANES (2017–2018) *n* = 1248, *N* = 152,470,957; NHANES (2015–2016) *n *= 1221, *N* = 149,646,377.

Partial least squares (PLS) regression was used to explore associations between multiple risk factors and each metal concentration [28]. Metal concentrations were natural log-transformed, and continuous risk factor variables were centered and scaled. PLS was selected because it accounts for large numbers of predictor variables and addresses potential multicollinearity among them. All risk factors were included in a PLS regression model. Urinary creatinine was also added as a covariate to all models to account for hydration status, which can affect urinary metal concentration. PLS reduces predictors into latent components that maximize covariance with the outcome while accounting for any collinearity. The optimal number of components for each metal-specific model was selected using cross-validation. Beta coefficients were estimated by regressing metal concentrations on the PLS components, representing weighted combinations of the predictors. Using bootstrapping, where the data were resampled with replacement 1000 times, the model was refitted to generate a distribution of coefficients, from which confidence intervals were derived. Beta coefficients were exponentiated and calculated as a percentage change in the urinary metal concentration for a one standard deviation increase in continuous variables, or in the group of interest compared with the reference group for binary variables. To assess the potential nonlinearity of age and urinary metal concentrations, we performed a sensitivity analysis by including an age-squared variable in the PLS model.

## Results

### Participant characteristics

Participant characteristics are summarized in Table 1. Women were, on average, younger (55.8 vs. 60.3 years) and more educated compared to men (20.0 vs 8.2% attended some university or higher. The mean BMI for women was 31.1, while the mean for men was 27.8. Very few women were current smokers (3.0%) or used alcohol (1.9%). Men walked over 2 h more per week than women, but the differences between men and women in television watching and sleep duration were small. Men’s diets included larger amounts in grams/day of fish, rice, and root vegetables compared with women's, while women’s diets included higher amounts of chicken and non-root vegetables.

**Table 1:** Participant characteristics by sex.

	Characteristic	Female	Male
	*N*	430	466
		Mean (SD) or *n* (%)	
Demographics	Age (years)	55.8 (8.8)	60.3 (5.0)
	Education Level Completed		
	Primary School	129 (30.0)	323 (69.3)
	Secondary School	215 (50.0)	105 (22.5)
	Some University or Higher	86 (20.0)	38 (8.2)
Anthropometry	BMI (kg/m^2^)	31.1 (5.7)	27.8 (4.4)
	Muscle Attenuation (mg/cm^3^)	71.2 (5.0)	72.5 (3.7)
Lifestyle	Smoking		
	Never	417 (97.0)	340 (73.0)
	Past/Current	13 (3.0)	126 (27.0)
	Alcohol (>4 units/week)		
	No	422 (98.1)	393 (84.3)
	Yes	8 (1.9)	73 (15.7)
Activity	Walking (hours/week)	1.0 (1.7)	3.2 (4.1)
	Television (>14 h/week)		
	No	215 (50.0)	239 (51.3)
	Yes	215 (50.0)	227 (48.7)
	Sleep (hours/night)	6.4 (1.5)	6.7 (1.4)
Diet	Food Frequency (g/day)		
	Fish	47.9 (45.0)	89.5 (74.0)
	Processed Fish	3.9 (7.5)	5.5 (9.4)
	Chicken	41.5 (47.7)	38.3 (37.9)
	Plain Rice	27.0 (39.5)	39.4 (54.4)
	Vegetable (Root)	81.2 (75.0)	97.2 (79.7)
	Vegetable (Non-Root)	484.5 (260.4)	443.6 (228.1)
	Legumes	108.7 (80.0)	110.3 (78.6)

### Urinary metal concentration summary and comparison with other populations

Six metals (Be, Cr, Mn, Pt, Sb, W) were not highly detected in either men or women (Table 2). All 12 of the highly detected metals (originally defined as >70% of participants with concentrations above LOD) were detected in more than 87% of participants, and 9 of the metals were detected in 100% of the participants (As, Cd, Co, Cs, Cu, Mo, Pb, Tl and Zn). Sex-stratified Spearman’s correlation coefficients of pairwise metals, as well as urinary creatinine, are shown in Fig. S2.

**Table 2:** Urinary Metal Concentration (μg/L) Summary for the Tobago Health Study.

	Metal	LOD	Sex	Percent Detected	GM	GSD	Min	10th Perc.	25th Perc.	50th Perc.	75th Perc.	90th Perc.	Max
Highly Detected (>70%)	As	0.61	Men	100	60.8	2.82	2.86	17.7	30.0	58.2	119	224	1850
			Women	100	35.7	2.98	1.57	9.60	15.6	34.8	77.3	151	496
	Ba	0.14	Men	98	1.16	2.63	<LOD	0.324	0.620	1.22	2.20	3.50	23.3
			Women	100	1.09	2.20	0.154	0.398	0.647	1.09	1.94	3.06	22.2
	Cd	0.01	Men	100	0.542	1.89	0.0621	0.257	0.366	0.514	0.814	1.18	5.38
			Women	100	0.735	2.10	0.0438	0.283	0.459	0.726	1.28	1.91	7.72
	Co	0.0078	Men	100	0.290	2.08	0.0181	0.140	0.185	0.268	0.408	0.632	14.9
			Women	100	0.427	2.59	0.0323	0.151	0.228	0.384	0.645	1.61	35.9
	Cs	0.19	Men	100	7.17	1.66	0.399	3.89	5.41	7.24	10.0	13.2	33.4
			Women	100	6.46	1.82	0.392	3.02	4.65	6.84	9.50	13.0	27.1
	Cu	0.48	Men	100	14.3	1.66	2.34	7.70	10.3	14.1	19.4	25.7	79.9
			Women	100	13.9	1.78	1.06	6.72	10.0	14.2	19.7	28.6	192
	Mo	1.2	Men	100	56.1	1.94	7.72	23.4	36.5	58.0	87.5	129	317
			Women	100	52.0	1.94	4.28	22.8	36.0	53.8	78.9	115	337
	Pb	0.09	Men	100	0.860	1.85	<LOD	0.402	0.599	0.865	1.27	1.83	9.15
			Women	100	0.857	1.73	0.105	0.436	0.600	0.863	1.19	1.63	7.88
	Sn	0.19	Men	94	0.779	3.10	<LOD	0.224	0.383	0.692	1.25	2.74	482
			Women	90	0.668	3.13	<LOD	<LOD	0.318	0.583	1.19	2.59	1190
	Tl	0.0057	Men	100	0.280	1.71	0.0101	0.147	0.203	0.288	0.400	0.520	1.06
			Women	100	0.249	1.77	0.0221	0.116	0.184	0.258	0.368	0.482	0.999
	U	0.0012	Men	87	0.0032	2.22	<LOD	<LOD	0.0018	0.0032	0.0053	0.00825	0.302
			Women	95	0.0039	1.99	<LOD	0.0015	0.0025	0.0039	0.0063	0.00941	0.0505
	Zn	4.7	Men	100	752	2.00	68.2	310	502	790	1160	1680	27300
			Women	100	627	2.24	35.9	221	395	644	1050	1790	4780
Not Highly Detected <=70%	Be	0.0057	Men	0	NC	NC	<LOD	<LOD	<LOD	<LOD	<LOD	<LOD	<LOD
			Women	0	NC	NC	<LOD	<LOD	<LOD	<LOD	<LOD	<LOD	<LOD
	Cr	0.41	Men	25	NC	NC	<LOD	<LOD	<LOD	<LOD	0.412	0.626	2.10
			Women	12	NC	NC	<LOD	<LOD	<LOD	<LOD	<LOD	0.431	3.10
	Mn	0.19	Men	30	NC	NC	<LOD	<LOD	<LOD	<LOD	0.213	0.348	7.49
			Women	65	0.269	2.05	<LOD	<LOD	<LOD	0.258	0.388	0.603	26.5
	Pt	0.0089	Men	3	NC	NC	<LOD	<LOD	<LOD	<LOD	<LOD	<LOD	0.0234
			Women	2	NC	NC	<LOD	<LOD	<LOD	<LOD	<LOD	<LOD	0.0261
	Sb	0.059	Men	18	NC	NC	<LOD	<LOD	<LOD	<LOD	<LOD	0.0858	0.295
			Women	15	NC	NC	<LOD	<LOD	<LOD	<LOD	<LOD	0.0733	0.626
	W	0.29	Men	1	NC	NC	<LOD	<LOD	<LOD	<LOD	<LOD	<LOD	0.659
			Women	1	NC	NC	<LOD	<LOD	<LOD	<LOD	<LOD	<LOD	0.615

When comparing the geometric means (GM) of THS metal concentrations to NHANES [26, 27] and other cross-sectional studies conducted in Africa, Asia, Europe, and North America [7, 18–25], THS men had a GM higher than 50% of the GMs reported in the comparison studies for every metal except Co and U, while women had higher GMs for every metal except Ba and U (Table 3). Notably, among THS men, GMs for Cs, Cu, Sn, Tl, and Zn exceeded the 75th percentile of the GMs from the comparison studies, whereas GMs for women’s Cd, Tl, and Zn concentrations in urine surpassed the 75th percentile reported in prior studies.

**Table 3:** Comparison of the Geometric Means of Tobago Health Study (THS) Urinary Metal Concentrations (μg/L) to Global Studies.

	Sex	Tobago Health Study	Puerto Rico^1^ [7]	US^2^ [26, 27]	Canada [18]	Ghana^3^ [19]	Germany [20]	Italy [21]	Ethiopia [22]	Bangladesh [23]	Indonesia [23]	Nepal [23]	Vietnam [23]	China [24]	Taiwan [25]
**n**		896	660	1248	6311	57	77	260	386	549	186	700	496	98	1871
**Ages**		40–87	18–40	40–87	3–79	19–60	19–78	18–60	10–50	18+	18+	18+	18+	35-85	18+
**As**	Men	60.80		4.59		94.33									72.90
	Women	35.70	10.90	4.11											70.90
	Both				8.90		9.20		20.90	85.00	53.10	26.20	107.00	20.10	
**Ba**	Men	1.16		1.10											
	Women	1.09	2.50	0.88											
	Both													1.69	
**Cd**	Men	0.54		0.21		0.38		0.24							0.59
	Women	0.74	0.12	0.22				0.27							0.64
	Both				0.38		0.16		0.61	0.79	0.51	0.39	0.94	0.34	
**Co**	Men	0.29		0.41		1.79		0.34							0.28
	Women	0.43	1.00	0.35				0.53							0.42
	Both				0.23		0.35		1.05						
**Cs**	Men	7.17		4.68											
	Women	6.46	4.90	3.75											
	Both				4.60									7.36	
**Cu**	Men	14.30				37.33		13.30							11.00
	Women	13.90	14.00					10.60							9.89
	Both				11.00				5.21						
**Mo**	Men	56.10		33.69											
	Women	52.00	58.90	25.50											
	Both				45.00		32.00		335.00						
**Pb**	Men	0.86		0.39		1.12		0.74							0.96
	Women	0.86	0.25	0.26				0.58							0.88
	Both				0.51		0.59		0.21	2.80	0.84	1.07	1.29	1.27	
**Sn**	Men	0.78		0.48											
	Women	0.67	2.10	0.48											
	Both						0.41								
**Tl**	Men	0.28		0.16				0.23							0.19
	Women	0.25		0.13				0.18							0.18
	Both				0.23		0.21		1.44						
**U**	Men	0.0032		0.0047											
	Women	0.0039		0.0055											
	Both														
**Zn**	Men	752.00				437.21									432.29
	Women	627.00	266.00												435.47
	Both				320.00		308.00		283.00					462.00	

^1^Ashrap et al. study includes pregnant women.

^2^includes NHANES 2017-2018 for all but one metal (*n* = 1248) and NHANES 2015-2016 for U only (*n* = 1211).

^3^Basu et al. study includes the gold mining community.

### Risk factors identified for urinary metal concentrations

**Fig. 1:**
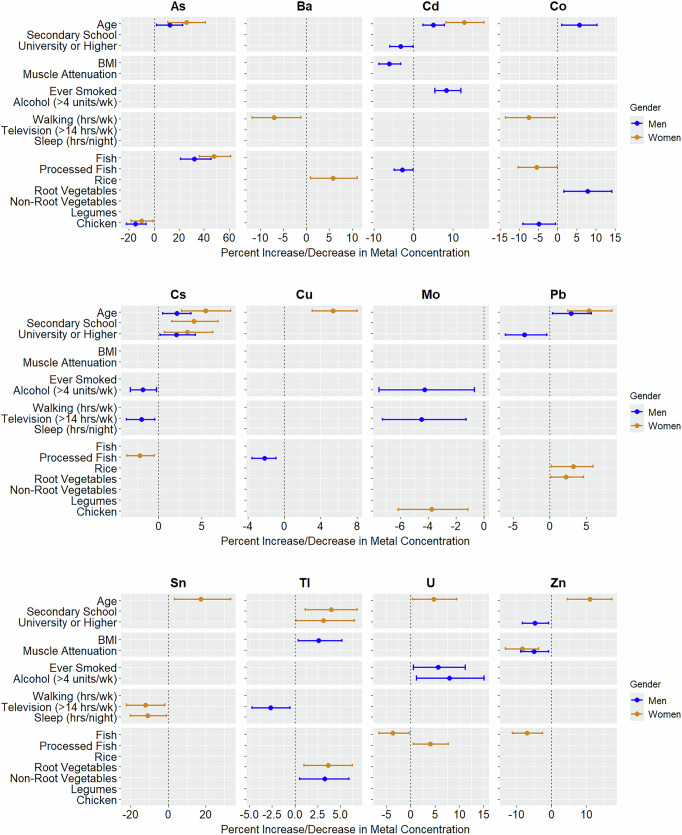
Forest plots with significant (p < 0.05) percentage change and 95% confidence intervals for log-transformed metals regressed on individual risk factors and log-transformed urinary creatinine are represented. For continuous risk factors, points reflect percentage change for a one standard deviation increase in the variable, while binary variables reflect percentage change in the group of interest compared to the reference group.

Results of the sex-stratified PLS regression are presented in Fig. 1. For women, a one SD increase in age (8.8 years) was associated with a higher concentration of eight metals, including As (26.2%), Sn (17.4%), Cd (12.8%), Zn (11.1%), Cs (5.5%), Cu (5.4%), Pb (5.4%), and U (4.7%). Attending secondary school and attending university or higher was associated with 4.1% and 3.3% higher Cs, respectively, and 4.0% and 3.1% higher Tl, respectively, compared to attending primary school only. A one SD increase in muscle attenuation (5.0 mg/cm^3^, in which higher values indicate lower intramuscular lipid infiltration) was associated with 8.4% lower Zn. A one SD increase in the time spent walking per week (1.7 h) was associated with lower Co (7.6%) and lower Ba (6.9%). Watching television for more than 14 h per week, and a one SD increase in sleep hours (1.5 h/night) was associated with 12.2% and 11.0% lower Sn, respectively. A one SD increase in daily fish consumption (45 g/day) was associated with higher As (48.0%), lower Zn (7.0%), and lower U (3.6%). A one SD increase in processed fish consumption (7.5 g/day) was associated with lower Co (5.5%) and Cs (2.2%) as well as higher U (4.0%) concentrations. A one SD increase in daily chicken consumption (47.7 g/day) was associated with 9.8% lower As and 3.8% lower Mo. A one SD increase in daily root vegetable consumption was associated with 3.7% higher Tl and 2.2% higher Pb. A one SD increase in daily rice consumption (39.5 g/day) was associated with higher Ba (5.7%) and Pb (3.2%).

For men, a one SD increase in age (5 years) was associated with higher As (12.7%), Co (5.7%), Cd (5.0%), as well as higher Pb and Cs ( < 3.0%). Attending university or higher, compared to attending primary school, was associated with lower Zn (4.8%), Pb (3.5%), and Cd (3.3%), and higher Cs (2.1%). A one SD increase in BMI (4.4 kg/m^2^) was associated with lower Cd (6.2%) and higher Tl (2.6%). A one SD increase in muscle attenuation (3.7 mg/cm^3^) was associated with 5.1% lower Zn. Current or past smoking was associated with 8.4% higher Cd and 5.6% higher U, while alcohol use was associated with 8.0% higher U, 4.5% lower Mo, and 1.8% lower Cs. In terms of physical activity, watching television for more than 14 h per week was associated with 4.3% lower Mo, 2.7% lower Tl, and 2.0% lower Cs. A one SD increase in daily fish consumption (74.0 g/day) was associated with 32.3% higher As, and processed fish consumption (9.4 g/day) was associated with lower Cd (2.8%) and lower Cu (2.2%). A one SD increase in root vegetable consumption (79.7 g/day) and non-root vegetable consumption (228.1 g/day) was associated with 7.9% higher Co and 3.3% higher Tl, respectively. Lastly, a one SD increase in chicken consumption (37.9 g/day) was associated with lower As (14.9%) and Co (4.9%).

In a sensitivity analysis assessing the potential nonlinearity of age by including an age-squared variable in the PLS model, neither age nor age-squared had a statistically significant association with any urinary metal concentrations. Additionally, confidence intervals were much wider for the age and age-squared variables when included together compared to the inclusion of the age variable alone, suggesting limited evidence for a nonlinear relationship between age and urinary metal concentrations within the age range of study participants.

The adjusted R-squared values for each metal model indicate the proportion of variance in log-transformed metal concentrations explained by the model, accounting for the number of variables included (Table S1). For men, the model explained 52% of the variance in Cs and Cd concentrations. Additionally, the model explained 37%, 36%, and 34% in Cu, Tl, and Zn concentrations, respectively. In contrast, it explained only 5% and 11% of the variance in Ba and Sn, suggesting that additional unmeasured factors may influence these metals in particular. Among women, the model explained 56% of the variance in Cs and 52% in Cu concentrations. Similar to men, it accounted for 37–43% of the variance in Cd, Tl, and Zn, as well as 39% in Mo. For all metals except Pb, Co, and Cd, the model explained a greater proportion of the variance in metal concentrations among women than among men.

## Discussion

Metal exposures in the Caribbean have not been well characterized, particularly in middle-aged and older adults. In this study, we (1) characterized metal exposure in Caribbean adults, and (2) identified risk factors, including personal characteristics, lifestyle behaviors, and dietary patterns. No prior study has comprehensively characterized urinary elements in Tobago. We found that these risk factors explained up to 52% of the variability in metal concentrations. Of the 18 metal concentrations measured, 12 were detected in more than 87% of the samples, and nine metals (including Co, Cu, Mo, and Zn, which are essential metals in some forms) were detected in all samples. Compared to NHANES and other international studies, THS men and women generally had higher geometric mean metal concentrations, with 5 elements in men (Cs, Cu, Sn, Tl, and Zn) and 3 in women (Cd, Tl, and Zn) exceeding the 75th percentile of values reported in the comparison studies.

There are a few other studies characterizing metal concentrations in Caribbean populations, aside from a study of pregnant women from Puerto Rico [7]. Beyond the Caribbean islands, there was a notable absence of studies measuring metal concentrations from other small island nations around the world. Only one study from China included middle-aged and older adults [24], while most others included young adults and sometimes included children as well. Not all metals were measured in all comparison studies. Comparison across studies was more challenging for certain metals, such as U, which was only measured in NHANES, and Sn, Ba, and Cs, which were included in fewer than four studies. In contrast, metals like Cd and Pb were measured in all studies, allowing for more consistent comparisons. Comparisons of urinary metal concentrations across countries should be interpreted with caution. Differences in age structure, timing of sample collection, and demographic composition (e.g., by education, socioeconomic status, and sex) can influence exposure distributions. Therefore, observed differences in metal levels reflect a combination of variation in exposure as well as in population characteristics. More human biomonitoring studies are needed, particularly those focused on middle-aged and older adults, and populations in the Caribbean and other small island nations, as well as those that measure a variety of metals.

The differing metal concentrations in the THS population compared to other studies may reflect regional factors such as differences in dietary habits and industrial activities in neighboring Trinidad and Venezuela. For example, higher seafood consumption typical in the Caribbean diet may contribute to higher As and Cd concentrations, as these metals can bioaccumulate in marine animals [29]. Additionally, soil and sediment contamination from industrial activities and agricultural runoff may contribute to higher concentrations of Cd and Pb [30]. Although some metal concentrations were higher in the THS population compared to others, this does not necessarily indicate that they surpass levels associated with adverse health effects. The lack of established safety thresholds for urinary metal concentrations underscores the need for population-specific reference ranges and further research to better understand the potential health implications of metal exposures.

Correlations between metal concentrations may suggest common sources and exposure pathways [31]. In this study, the strongest correlation was between Tl and Cs in women, although we found no research suggesting a possible common source for these two metals. In contrast, the weak correlations of As and Ba with other metals may suggest distinct exposure sources or differences in the metabolism and excretion of these two metals compared to all others. Women had more correlated metal concentrations compared with men, although they exhibited the same pattern of correlation, which again may suggest common sources for men and women, but more sources of variation in metal concentrations for men.

Differences in variation between men and women were reflected in the adjusted R-squared values from the PLS models, which were generally lower in men than in women for all metals except Cd and cobalt Co. This suggests that the measured risk factors accounted for more of the variation in metal exposures among women, potentially due to sex differences in exposure sources, behaviors, or biological processing. Overall, the models explained only a modest proportion of the variation for some metals, such as Ba and Sn, and more for others, like Cs and Cd. These findings indicate that unmeasured environmental or behavioral factors likely contribute to the variation in metal concentrations, underscoring the need for future research to identify additional risk factors and sources of exposure. In particular, nutritional status and supplementation, body composition and chronic disease status, as well as reproductive histories for women, should be examined as additional risk factors for metal exposures, metabolism and excretion in future studies.

Increased urinary metal concentrations were associated with various demographic, lifestyle, and dietary factors, with notable differences in the specific risk factors linked to metal concentrations between men and women. These differences may reflect biological variations in metal absorption, distribution, metabolism, and excretion [32], as well as differences in exposure sources, such as occupational roles that vary between Caribbean men and women [33]. Additionally, there were some notable demographic differences between men and women, including age and education. Initially, male-only participants were recruited in 1999 for a prostate cancer study. Some additional male participants were recruited and continued to be followed in subsequent study visits. Urine samples for metals measurement were collected at the sixth follow-up visit for the men’s study (V6_men_: 2014–2016), and at the baseline visit for the women’s study (V1_women_: 2019–2020), which contributed to the different age distribution between men and women despite recruitment protocols being identical for men and women. In addition to women having a younger age distribution, which may partially explain the higher educational attainment of women, Caribbean women generally have higher educational attainment compared with men [34].

Older age was associated with higher concentrations of As, Cd, Cs, and Pb in both men and women. Older age is typically associated with higher concentrations of Cd and Pb, as these metals accumulate in the body over time due to their long biological half-lives, which can span decades [35]. Additionally, increased bone turnover associated with aging may mobilize stored Pb, leading to higher urinary concentrations [36].

Cd, Pb, and Zn were lower in men who attended university compared to men who attended primary school only. Educational attainment was included as a proxy for socio-economic position, as it can represent social opportunities and cultural capital [37]. Socio-economic position may affect metal and other environmental toxicant exposures in several ways, including the location and quality of housing, occupation, diet, and other health-related behaviors [38, 39]. Higher educational attainment was associated with higher Cs in both men and women and higher Tl in women only, indicating that there may be some exposures associated with access to more resources, such as travel, the use of personal care products, or imported foods [17, 40, 41]. Alternatively, higher urine concentrations in individuals with higher educational attainment may also be due to increased excretion of these metals as opposed to increased exposures compared to participants with lower educational attainment.

In this study, many lifestyle factors were also associated with metal concentrations. Current and former smoking was associated with higher Cd and U concentrations in men, which is consistent with the established evidence that tobacco smoke contains various metals, and that tobacco smoke is typically the main source of Cd for those who smoke or are exposed to second-hand smoke [42]. Television viewing time, a proxy variable for sedentary behavior, was associated with lower Cs, Mo, and Tl in men and Sn in women. Conversely, for women, longer walking time per week was associated with lower Ba and Co. Physical activity affects the metabolism of environmental toxicants, such as persistent organic pollutants, either through preventing or correcting for bioaccumulation [43]. The effect of physical activity on metal metabolism, however, has not been well studied. Lastly, longer sleep duration was associated with lower Sn for women. Exposure to air pollutants has been found to be associated with longer sleep duration; the effect of non-essential metals on sleep has not been well studied either [44].

Foods including fish, chicken, rice, and both root and non-root vegetables were associated with higher concentrations of As, Ba, Co, Pb, Tl, and U. These metals can bioaccumulate in plant and animal tissues, leading to higher exposure at ingestion [45]. Seafood is a well-known source of As exposure, primarily in the form of organic arsenic, which is generally considered less toxic, but it can also contribute to inorganic As exposure. Two shark species, which are consumed in Trinidad and Tobago, had concentrations of As, Cd, Pb, and Hg that posed significant health risks to consumers [29]. Beyond the metal content in foods, nutritional status may affect the toxicokinetics of metals within the body [46]. One study investigated diet patterns with a multi-ethnic population in Tobago [47]. While nutrient deficiencies were rare, some women had deficiencies in specific vitamins, minerals, and essential metals, which can affect the absorption, distribution, metabolism, and excretion of toxic metals. Some foods were associated with lower urine concentrations of metals. These inverse relationships may reflect differences in dietary patterns, whereby greater intake of one food substitutes for other foods that are stronger sources of these metals. Nutrient interactions, such as selenium or calcium, provided by certain foods may alter the metabolism and excretion of some metals [46]. More research is needed to understand the complex relationships between dietary patterns and urinary metal concentrations.

Higher BMI was associated with lower concentrations of Cd and higher Tl for men. Metal exposure is associated with obesity and metabolic syndrome, with studies demonstrating that low doses of metal exposure may stimulate adipogenesis while higher doses inhibit adipocyte differentiation, leading to metabolic disturbances [48]. The inverse relationship between some metals and BMI may be the result of increased sequestration of metals like Cd in adipose tissue, or altered excretion due to a higher risk of inflammation and kidney dysfunction associated with obesity [49]. Lastly, muscle attenuation was associated with lower Zn in both men and women. Zn deficiency is associated with altered body composition, including increased fat infiltration in muscle tissue [50]. Essential metals are tightly regulated in the body; however, chronic conditions can disrupt homeostasis, leading to excess urinary excretion. Specifically, individuals with type 2 diabetes or metabolic syndrome have higher urinary excretion of Cu and Zn compared to those without these conditions [51]. It is possible that there is a bidirectional relationship between metals and body composition or other metabolic conditions. More research is needed to disentangle the complex relationships between metal exposures, metabolism, excretion and metabolic outcomes.

There are some challenges to using urine as a medium for biomonitoring. Some metals can be stored within the body for longer periods, causing excretion to be inconsistent over time [52]. For example, Pb can be stored long-term in bone and may be released during times of high bone turnover, such as pregnancy or age-related bone loss [36]. Due to the long duration of Pb in the body, it is difficult to determine the exposure window for Pb in urine [53]. Similarly, Cd can accumulate in the proximal tubules of the kidney, which may impact its excretion as well as the excretion of other metals [54]. Despite these challenges, urine remains a valuable tool for biomonitoring. Urine is a non-invasive and cost-effective way to collect biological samples in larger-scale population studies and for repeated sampling to measure changes over time. Lastly, urinary metal concentrations for essential metals can provide insights into chronic conditions that disrupt homeostasis and lead to excess excretion.

This study had several limitations. This study is cross-sectional, which makes causality difficult to determine. Urine samples for men and women were collected in different years (2014–2016 for men and 2019–2020 for women). It is difficult to determine if the observed differences in urinary metal concentrations between men and women are due to temporal differences in urine collection and/or due to other reasons, such as differences in exposures or metal toxicokinetics. To our knowledge, there are no published longitudinal or repeated cross-sectional studies from Tobago or comparable Caribbean populations that characterize temporal trends in metal exposures over this or any other period. In longitudinal studies from other regions, whether metal exposures decreased over time was metal-specific (e.g., As, Pb) and used to identify potential targets for exposure mitigation interventions [55–60]. The findings of this study can serve as a benchmark for this cohort, and measurement of subsequent samples for metals would allow the assessment of exposure trajectories, emerging metal sources, and changes in exposure-related health outcomes. Additionally, more women had urine samples with insufficient volume for metal measurement compared to men. Despite the missing data for women, we do not have reason to believe that women with insufficient sample volume differed significantly from those with adequate sample volumes. Lastly, self-reported diet and physical activity data were collected via interview and may be subject to recall and social-desirability bias. It is important to note that our findings may not be generalizable to younger men and women from Tobago, nor to populations of other Caribbean Islands, where environmental exposures may differ considerably.

This study also had several strengths. To our knowledge, this is the first study to characterize metal concentrations in a population of middle-aged and older African Caribbean adults from Tobago. This subsample was randomly selected from the larger THS cohort, which is a well-established population-based prospective cohort with extensive measures of lifestyle variables, increasing the representativeness of the results among African Caribbean adults from Tobago. The study has a large sample size and a relatively balanced representation of men and women, allowing for some insight into potential differences in exposure patterns or risk factors in men and women. Lastly, the New York State Department of Health’s Wadsworth Center, which measured metal concentrations, used rigorous internal and external QA/QC protocols and well-validated laboratory methods to ensure the integrity of the results.

This study can inform future research into metal exposures and the associated health impacts for Tobagonian adults. In particular, an investigation into the source of the differences in metal concentrations for men and women is needed. Additionally, research on the modifiable risk factors like physical activity and diet that affect metal concentrations can be further investigated to inform strategies for both exposure prevention and mitigation of metal-related adverse health outcomes.

## Supplementary information

Supplementary material

## Data Availability

Metal concentration data for this analysis are hosted and available through the Human Health Exposure Resource (HHEAR) Data Repository (https://hheardatacenter.mssm.edu), which has been approved under Icahn School of Medicine at Mount Sinai IRB Protocol #16-00947. Access to other study population data is available upon request, pending submission of an analysis plan and approval by the Tobago Health Study.

## References

[1] Vareda JP, Valente AJM, Durães L. Assessment of heavy metal pollution from anthropogenic activities and remediation strategies: a review. J Environ Manag. 2019;246:101–18. 10.1016/j.jenvman.2019.05.126 31176176

[2] Piwowarska D, Kiedrzyńska E, Jaszczyszyn K. A global perspective on the nature and fate of heavy metals polluting water ecosystems, and their impact and remediation. Crit Rev Environ Sci Technol. 2024;54:1436–58.

[3] Haidar Z, Fatema K, Shoily S, Sajib A. Disease-associated metabolic pathways affected by heavy metals and metalloids. Toxicol Rep. 2023;10:554–70. 10.1016/j.toxrep.2023.04.010 PMC10313886 37396849

[4] Lamas G, Bhatnagar A, Jones M, Mann K, Nasir K, Tellez-Plaza M, et al. Contaminant metals as cardiovascular risk factors: a scientific statement from the American Heart Association. J Am Heart Assoc. 2023;12:e029852. 10.1161/JAHA.123.029852 PMC10356104 37306302

[5] Bordon IC Metals and new approaches for biomonitoring. Integr Environ Assess Manag. 2023;19:1411–3. 10.1002/ieam.4841 37850523

[6] Tamayo-Ortiz M, Riojas-Rodríguez H, Téllez-Rojo MM, Boischio A, Mañay N, Menezes-Filho JA, et al. A call for biomonitoring systems in Latin America and the Caribbean: considerations for potentially toxic metals/metalloids. Ann Glob Health. 2022;88:80. 36185997 10.5334/aogh.3637 PMC9479654

[7] Ashrap P, Watkins DJ, Mukherjee B, Boss J, Richards MJ, Rosario Z, et al. Predictors of urinary and blood Metal(loid) concentrations among pregnant women in Northern Puerto Rico. Environ Res. 2020;183:109178. 32007748 10.1016/j.envres.2020.109178 PMC7167342

[8] Risher J, Todd GD, Meyer D, Zunker CL. The elderly as a sensitive population in environmental exposures: making the case. Rev Environ Contam Toxicol. 2010;207:95–157. 10.1007/978-1-4419-6406-9_2 20652665

[9] Bunker CH, Patrick AL, Konety BR, Dhir R, Brufsky AM, Vivas CA, et al. High prevalence of screening-detected prostate cancer among Afro-Caribbeans: the Tobago Prostate Cancer Survey. Cancer Epidemiol Biomarkers Prev. 2002;11:725–9. 12163325

[10] Acevedo-Fontánez A, Cvejkus R, Zmuda J, Kuipers A, Barinas-Mitchell E, Sekikawa A, et al. Skeletal muscle adiposity is a novel risk factor for poor cognition in African-Caribbean women. Obesity. 2023;31:2398–2406. 10.1002/oby.23816 PMC10680092 37475604

[11] Kuipers AL, Miljkovic I, Barinas-Mitchell E, Cvejkus R, Bunker CH, Wheeler VW, et al. Arterial stiffness and hypertension status in Afro-Caribbean men. J Hypertens. 2019;37:546–54. 30234778 10.1097/HJH.0000000000001909 PMC6355357

[12] Galusha AL, Merrill L, Palmer CD, Amarasiriwardena C, Parsons PJ. Measurement harmonization and traceability for trace element analyses across the Children’s Health Exposure Analysis Resource laboratory network. Environ Res. 2021;193:110302. 10.1016/j.envres.2020.110302 PMC8924990 33049243

[13] Centers for Disease Control and Prevention, National Center for Health Statistics. NHANES 2017-2018: metals - urine (UM_J) data documentation, codebook, and frequencies [Internet]. Atlanta (GA): CDC; 2021 [cited 2025 May 14]. Available from: https://wwwn.cdc.gov/Nchs/Data/Nhanes/Public/2017/DataFiles/UM_J.htm

[14] Schisterman EF, Little RJ. Opening the black box of biomarker measurement error. Epidemiology. 2010;21 Suppl 4(Suppl 4):S1-3. 10.1097/EDE.0b013e3181dda514 PMC3042241 20539119

[15] Kannan K, Stathis A, Mazzella MJ, Andra SS, Barr DB, Hecht SS, et al. Quality assurance and harmonization for targeted biomonitoring measurements of environmental organic chemicals across the Children’s Health Exposure Analysis Resource laboratory network. Int J Hyg Environ Health. 2021;234:113741. 10.1016/j.ijheh.2021.113741 PMC8096700 33773388

[16] Barr D, Wilder L, Caudill S, Gonzalez A, Needham L, Pirkle J. Urinary creatinine concentrations in the U.S. population: implications for urinary biologic monitoring measurements. Environ Health Perspect. 2005;113:192–200. 10.1289/ehp.7337 PMC1277864 15687057

[17] Scutarașu EC, Trincă LC. Heavy metals in foods and beverages: global situation, health risks and reduction methods. Foods. 2023;12:3340. 10.3390/foods12183340 PMC10528236 37761050

[18] Haines DA, Saravanabhavan G, Werry K, Khoury C. An overview of human biomonitoring of environmental chemicals in the Canadian Health Measures Survey: 2007–2019. Int J Hyg Environ Health. 2017;220:13–28. 10.1016/j.ijheh.2016.08.002 27601095

[19] Basu N, Nam D-H, Kwansaa-Ansah E, Renne EP, Nriagu JO. Multiple metals exposure in a small-scale artisanal gold mining community. Environ Res. 2011;111:463–7. 10.1016/j.envres.2011.02.006 21397224

[20] Schmied A, Murawski A, Kolossa-Gehring M, Kujath P. Determination of trace elements in urine by inductively coupled plasma-tandem mass spectrometry—Biomonitoring of adults in the German capital region. Chemosphere. 2021;285:131425. 10.1016/j.chemosphere.2021.131425 34246933

[21] Aprea MC, Apostoli P, Bettinelli M, Lovreglio P, Negri S, Perbellini L, et al. Urinary levels of metal elements in the non-smoking general population in Italy: SIVR study 2012–2015. Toxicol Lett. 2018;298:177–85. 10.1016/j.toxlet.2018.07.004 30003948

[22] Godebo TR, Paul CJ, Jeuland MA, Tekle-Haimanot R. Biomonitoring of metals and trace elements in urine of central Ethiopian populations. Int J Hyg Environ Health. 2019;223:410–8. 10.1016/j.ijheh.2018.12.007 PMC6440854 30612877

[23] Mizuno Y, Shimizu-Furusawa H, Konishi S, Inaoka T, Ahmad SA, Sekiyama M, et al. Associations between urinary heavy metal concentrations and blood pressure in residents of Asian countries. Environ Health Prev Med. 2021;26:101. 34625018 10.1186/s12199-021-01027-y PMC8501740

[24] Li A, Mei Y, Zhao M, Xu J, Zhao J, Zhou Q, et al. Do urinary metals associate with the homeostasis of inflammatory mediators? Results from the perspective of inflammatory signaling in middle-aged and older adults. Environ Int. 2022;163:107237. 10.1016/j.envint.2022.107237 35429917

[25] Liao K-W, Chen P-C, Chou W-C, Shiue I, Huang H-I, Chang W-T, et al. Human biomonitoring reference values, exposure distribution, and characteristics of metals in the general population of Taiwan: Taiwan environmental survey for Toxicants (TESTs), 2013–2016. Int J Hyg Environ Health. 2023;252:114195. 10.1016/j.ijheh.2023.114195 37321161

[26] Centers for Disease Control and Prevention (CDC) National Center for Health Statistics (NCHS). National Health and Nutrition Examination Survey Data Hyattsville, MD: U.S. Department of Health and Human Services, Centers for Disease Control and Prevention; 2015–2016. Available from https://wwwn.cdc.gov/nchs/nhanes/continuousnhanes/default.aspx?BeginYear=2015

[27] Centers for Disease Control and Prevention (CDC) National Center for Health Statistics (NCHS). National Health and Nutrition Examination Survey Data Hyattsville, MD: U.S. Department of Health and Human Services, Centers for Disease Control and Prevention; 2017–2018. Available from https://wwwn.cdc.gov/nchs/nhanes/continuousnhanes/default.aspx?BeginYear=2017

[28] Wold H. Partial least squares. In: S. Kotz, C.B. Read, N. Balakrishnan, B. Vidakovic and N.L. Johnson, editors. Encyclopedia of statistical sciences. Second Edition. Hoboken, NJ, USA: John Wiley & Sons, Inc; 2006.

[29] Mohammed A, Mohammed T. Mercury, arsenic, cadmium, and lead in two commercial shark species (Sphyrna lewini and Caraharinus porosus) in Trinidad and Tobago. Mar Pollut Bull. 2017;119:214–8. 28438339 10.1016/j.marpolbul.2017.04.025

[30] Nelson W. Fractionation of trace metals in coastal sediments from Trinidad and Tobago, West Indies. Mar Pollut Bull. 2020;150:110774. 31785846 10.1016/j.marpolbul.2019.110774

[31] Chen C, Yuan T, Shie R, Wu K, Chan C. Linking sources to early effects by profiling the urine metabolome of residents living near oil refineries and coal-fired power plants. Environ Int. 2017;102:87–96. 10.1016/j.envint.2017.02.003 28238459

[32] Gochfeld M. Sex Differences in human and animal toxicology. Toxicol Pathol. 2016;45:11–28. 27895264 10.1177/0192623316677327 PMC5371029

[33] Ruprah IJ, Schimanski C, Chagalj C. Gender-based educational and occupational segregation in the Caribbean. Washington, DC: Inter-American Development Bank;2018. Report No.:IDB-WP-931. Available from: https://webimages.iadb.org/publications/english/document/Gender-based-Educational-and-Occupational-Segregation-in-the-Caribbean.pdf

[34] Miller E. Gender and democratization of Caribbean education. Caribb J Educ Dev. 2024;1:5–29.

[35] Agency for Toxic Substances Disease Registry. Toxicological profile for Cadmium, Atlanta, GA: U.S. Department of Health and Human Services, Public Health Service; 2012. Available from https://wwwn.cdc.gov/TSP/ToxProfiles/ToxProfiles.aspx?id=48&tid=15

[36] Collin M, Venkataraman Sk, Vijayakumar N, Kanimozhi V, Arbaaz S, Stacey RGS, et al. Bioaccumulation of lead (Pb) and its effects on human: a review. J Hazard Mater Adv. 2022;7:100094.

[37] Khalatbari-Soltani S, Maccora J, Blyth FM, Joannès C, Kelly-Irving M. Measuring education in the context of health inequalities. Int J Epidemiol. 2022;51:701–8. 10.1093/ije/dyac058 PMC9189977 35362521

[38] Evans G, Kantrowitz E. Socioeconomic status and health: the potential role of environmental risk exposure. Annual Rev Public Health. 2002;23:303–31. 10.1146/annurev.publhealth.23.112001.112349 11910065

[39] Montazeri P, Thomsen C, Casas M, de Bont J, Haug L, Maitre L, et al. Socioeconomic position and exposure to multiple environmental chemical contaminants in six European mother-child cohorts. Int J Hyg Environ Health. 2019;222:864–72. 10.1016/j.ijheh.2019.04.002 PMC8713641 31010791

[40] Arshad H, Mehmood MZ, Shah MH, Abbasi AM. Evaluation of heavy metals in cosmetic products and their health risk assessment. Saudi Pharm J 2020;28:779–90. 10.1016/j.jsps.2020.05.006 PMC7335825 32647479

[41] Poom A, Willberg E, Toivonen T. Environmental exposure during travel: a research review and suggestions forward. Health Place. 2021;70:102584. 10.1016/j.healthplace.2021.102584 34020232

[42] Pinto E, Cruz M, Ramos P, Santos A, Almeida A. Metals transfer from tobacco to cigarette smoke: evidence in smokers’ lung tissue. J Hazard Mater. 2017;325:31–5. 10.1016/j.jhazmat.2016.11.069 27914289

[43] Serrano QA, Le Garf S, Martin V, Colson SS, Chevalier N. Is physical activity an efficient strategy to control the adverse effects of persistent organic pollutants in the context of obesity? A narrative review. Int J Mol Sci. 2024;25:883. 38255955 10.3390/ijms25020883 PMC10815489

[44] Liu J, Ghastine L, Um P, Rovit E, Wu T. Environmental exposures and sleep outcomes: a review of evidence, potential mechanisms, and implications. Environ Res. 2021;196:110406. 10.1016/j.envres.2020.110406 PMC8081760 33130170

[45] Guerrieri N, Mazzini S, Borgonovo G. Food plants and environmental contamination: an update. Toxics. 2024;12:365. 10.3390/toxics12050365 PMC11125986 38787144

[46] Peraza M, Ayala-Fierro F, Barber D, Casarez E, Rael L. Effects of micronutrients on metal toxicity. Environ Health Perspect. 1998;106 Suppl 1:203–16. 10.1289/ehp.98106s1203 PMC1533267 9539014

[47] Nichols S, Dalrymple N, Prout P, Ramcharitar-Bourne A. Dietary intake patterns, nutrient adequacy and associated factors in a multi-ethnic Caribbean population. Nutr Health. 2023;29:297–307. 35014896 10.1177/02601060211070907

[48] Tinkov AA, Aschner M, Ke T, Ferrer B, Zhou J-C, Chang J-S, et al. Adipotropic effects of heavy metals and their potential role in obesity. Faculty Rev. 2021;10:32. 10.12703/r/10-32 PMC8103910 33977285

[49] Attia SM, Varadharajan K, Shanmugakonar M, Das SC, Al-Naemi HA, Attia SM, et al. Cadmium: an emerging role in adipose tissue dysfunction. Expo Health. 2021;14:171–83.

[50] Cunha TA, Vermeulen-Serpa KM, Grilo EC, Leite-Lais L, Brandão-Neto J, Vale SHL. Association between zinc and body composition: an integrative review. J Trace Elem Med Biol. 2022;71:126940. 10.1016/j.jtemb.2022.126940 35121408

[51] Galvez-Fernandez M, Powers M, Grau-Perez M, Domingo-Relloso A, Lolacono N, Goessler W, et al. Urinary zinc and incident type 2 diabetes: prospective evidence from the strong heart study. Diabetes Care. 2022;45:2561–9. 36134919 10.2337/dc22-1152 PMC9679259

[52] Elder A, Nordberg G, Kleinman M. Chapter 3 - Exposure, dose, and toxicokinetics of metals. In: L Friberg, VB Vouk, editors. Handbook on the toxicology of metals: Volume I: General Considerations. Fifth Edition. Elsevier; 2022:55–86.

[53] Martinez-Morata I, Sobel M, Tellez-Plaza M, Navas-Acien A, Howe CG, Sanchez TR. A state-of-the-science review on metal biomarkers. Curr Environ Health Rep. 2023;10:215–49. 10.1007/s40572-023-00402-x PMC10822714 37337116

[54] Satarug S, Vesey DA, Gobe GC, Đorđević AB. The validity of benchmark dose limit analysis for estimating permissible accumulation of cadmium. Int J Environ Res Public Health. 2022;19:15697. 10.3390/ijerph192315697 PMC9736539 36497771

[55] Becker K, Schroeter-Kermani C, Seiwert M, Rüther M, Conrad A, Schulz C, et al. German health-related environmental monitoring: Assessing time trends of the general population’s exposure to heavy metals. Int J Hyg Environ Health. 2013;216:250–4. 23410801 10.1016/j.ijheh.2013.01.002

[56] Ahn J, Kim N-S, Lee B-K, Oh I, Kim Y. Changes of atmospheric and blood concentrations of lead and cadmium in the general population of South Korea from 2008 to 2017. Int J Environ Res Public Health. 2019;16:2096. 31200504 10.3390/ijerph16122096 PMC6617041

[57] Abass K, Emelyanova A, Rautio A. Temporal trends of contaminants in Arctic human populations. Environ Sci Pollut Res Int. 2018;25:28834–50. 30145756 10.1007/s11356-018-2936-8 PMC6592971

[58] Lieberman-Cribbin W, Li Z, Lewin M, Ruiz P, Jarrett JM, Cole SA, et al. The contribution of declines in blood lead levels to reductions in blood pressure levels: longitudinal evidence in the Strong Heart Family Study. J Am Heart Assoc. 2024;13:e031256. 38205795 10.1161/JAHA.123.031256 PMC10926826

[59] Navas-Acien A, Umans JG, Howard BV, Goessler W, Francesconi KA, Crainiceanu CM, et al. Urine arsenic concentrations and species excretion patterns in American Indian communities over a 10-year period: the Strong Heart Study. Environ Health Perspect. 2009;117:1428–33. 19750109 10.1289/ehp.0800509 PMC2737021

[60] Wang Q, Wu J, Dong X, Niu W. Trends in urine lead and associated mortality in US adults: NHANES 1999–2018. Front Nutr. 2024;11:1411206. 38873569 10.3389/fnut.2024.1411206 PMC11169937

