# Supplementary material for: Risk factors associated with urinary metal concentrations in middle-aged and older Caribbean adults: the Tobago Health Study
Source: J Expo Sci Environ Epidemiol. 2026 Mar 30;36(4):777–87. doi: 10.1038/s41370-026-00860-z (PMC13331741; doi:10.1038/s41370-026-00860-z)
Supplement: Supplementary file 1 — Supplementary material [file 41370_2026_860_MOESM1_ESM.docx]

**Supplementary material**

**Table S1.** Adjusted R^2^ for the partial least squares models for each metal among men and women, assessing how well the model explains the variation in each metal concentration, adjusted by the number of variables included.

|  | Men Adjusted-R^2^ | Women Adjusted-R^2^ |
| --- | --- | --- |
| As | 13.3% | 23.3% |
| Ba | 4.7% | 6.0% |
| Cd | 51.6% | 42.9% |
| Co | 26.3% | 21.7% |
| Cs | 51.6% | 56.4% |
| Cu | 37.1% | 51.9% |
| Mo | 31.7% | 38.7% |
| Pb | 33.6% | 21.3% |
| Sn | 10.6% | 12.2% |
| Tl | 35.5% | 39.7% |
| U | 19.9% | 23.7% |
| Zn | 34.0% | 37.4% |


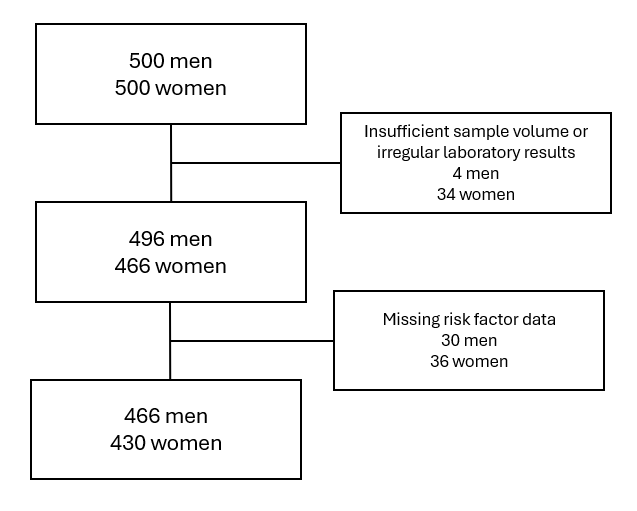


**Figure S1. Study Participant Flow Diagram**

(A)


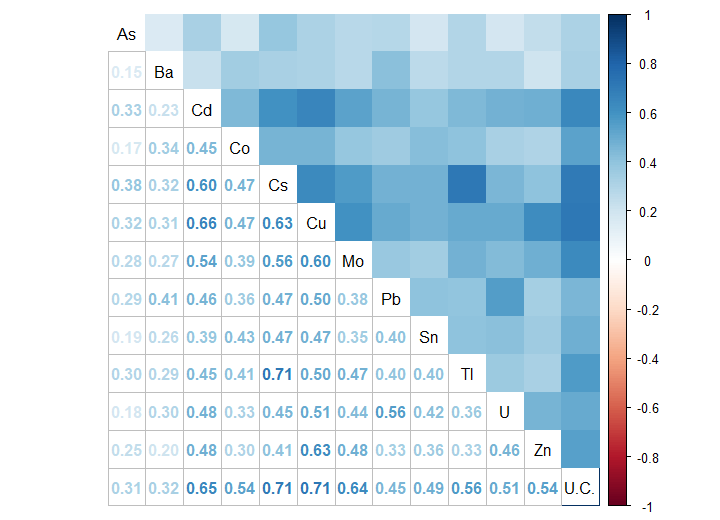


(B)

**
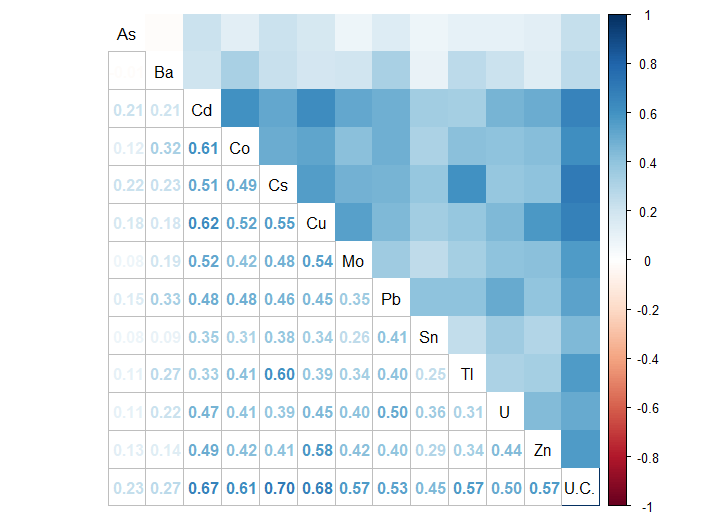
**

**Figure S2**. Spearman’s correlation plot for each pairwise metal and including urinary creatinine (U.C.) for women **(A)** and men **(B)**. For women, a few metal pairs were strongly correlated (0.6<ρ<0.7) including Tl and Cs, as well as Cu individually with Cs, Zn and Cd. As and Ba were weakly correlated with all other metals. Urinary creatinine in women was strongly correlated (0.6< ρ <0.7) with Cd, Cs, Cu and Mo and moderately correlated (0.4< ρ <0.6) with all the remaining metals except for As and Ba. For men there was a similar pattern of more highly correlated metal pairs compared with women, however, the correlation was generally weaker in men than in women, with only Cu and Cd being strongly correlated, as well as urinary creatinine with Cd, Co, Cs, and Cu.
